# O-GlycNacylation Remission Retards the Progression of Non-Alcoholic Fatty Liver Disease

**DOI:** 10.3390/cells11223637

**Published:** 2022-11-16

**Authors:** Yicheng Zhou, Zhangwang Li, Minxuan Xu, Deju Zhang, Jitao Ling, Peng Yu, Yunfeng Shen

**Affiliations:** 1 Department of Endocrinology and Metabolism, the Second Affiliated Hospital of Nanchang University, Branch of Nationlal Clinical Research Center for Metabolic Diseases, Institute for the Study of Endocrinology and Metabolism in Jiangxi Province, Nanchang 330006, China; 2 The Second Clinical Medical College of Nanchang University, Nanchang 330031, China; 3 Food and Nutritional Sciences, School of Biological Sciences, The University of Hong Kong, Pokfulam Road, Hong Kong

**Keywords:** O-GlcNAcylation, insulin resistance, non-alcoholic fatty liver disease, non-alcoholic steatohepatitis, fibrosis, cirrhosis, hepatocellular carcinoma

## Abstract

Non-alcoholic fatty liver disease (NAFLD) is a metabolic disease spectrum associated with insulin resistance (IR), from non-alcoholic fatty liver (NAFL) to non-alcoholic steatohepatitis (NASH), cirrhosis, and hepatocellular carcinoma (HCC). O-GlcNAcylation is a posttranslational modification, regulated by O-GlcNAc transferase (OGT) and O-GlcNAcase (OGA). Abnormal O-GlcNAcylation plays a key role in IR, fat deposition, inflammatory injury, fibrosis, and tumorigenesis. However, the specific mechanisms and clinical treatments of O-GlcNAcylation and NAFLD are yet to be elucidated. The modification contributes to understanding the pathogenesis and development of NAFLD, thus clarifying the protective effect of O-GlcNAcylation inhibition on liver injury. In this review, the crucial role of O-GlcNAcylation in NAFLD (from NAFL to HCC) is discussed, and the effect of therapeutics on O-GlcNAcylation and its potential mechanisms on NAFLD have been highlighted. These inferences present novel insights into the pathogenesis and treatments of NAFLD.

## 1. Introduction

Non-alcoholic fatty liver disease (NAFLD) is a clinicopathological syndrome with excessive fat deposition in the hepatocytes [1]. It is closely associated with metabolic syndrome, obesity, insulin resistance (IR), and dyslipidemia. Due to the lack of obvious symptoms and starting with simple steatosis in most NAFLD patients, the disease is missed. However, a subset of NAFLD can develop into non-alcoholic steatohepatitis (NASH), and 20% of NASH patients progress to hepatic fibrosis. Once fibrosis occurs, a poor prognosis is developed, such as liver cirrhosis or hepatocellular carcinoma (HCC), the second-most common cause of cancer-related deaths [2]. Nowadays, with the abrupt rising of obesity and diabetes worldwide, the incidence of NAFLD has escalated rapidly [3], with a global prevalence of 25% [4]. Metabolic abnormalities are closely associated with NAFLD, and it was, hence, renamed “metabolic dysfunction-associated fatty liver disease” (MAFLD) in 2020 [5]. (For the convenience of its description, this article has used NAFLD). Moreover, NAFLD is associated with a metabolic imbalance in glucose, lipids, amino acids, bile acids, and iron [6]. Several recent studies have focused on the role of glucose or other metabolisms in NAFLD. Among these, hyperglycemia is a major influencing factor on NAFLD and stimulates insulin secretion and increases the synthesis of triglycerides in the liver. The excessive triglycerides accumulate gradually in the liver and are exported to generate hypertriglyceridemia [7]. In addition, long-term and chronic hyperglycemia-induced hepatocytes injury alters the structure and function of pancreatic β-cells and causes IR, thereby inducing and accelerating the occurrence and progression of NAFLD [8]. Glucose and fructose are the primary mediators of NAFLD, leading to triglyceride production [9]. Therefore, it is of great significance to elucidate the pathogenesis of NAFLD.

One hypothesis of NAFLD pathogenesis has been described by the “2-hit theory” [10], whereby the first hit of hepatic triglyceride accumulation (hepatic steatosis) is induced by IR facilitated by the liver metabolism of fructose. In the second hit, fructose promotes the fructosylation of proteins, the formation of reactive oxygen species (ROS), due to the molecular instability of its five-membered furanose ring [8], endoplasmic reticulum (ER) stress, and inflammation [11], which causes hepatocellular damage and eventually fibrosis [12]. Gradually, the “2-hit theory” has been modified into the “multiple parallel hits” hypothesis for NASH pathogenesis, suggesting that liver damage is caused by multiple parallel pathogenic events [13]. Recently, glycosylation, a posttranslational modification of the proteins in glucose metabolism, has been under intensive focus. The N-glycosylation on the specific peptide sites of serum proteins is a potential marker for the diagnosis of NAFLD-associated hepatocellular carcinoma (NAFLD-HCC) [14]. In addition, the N-glycosylation of the cyclic adenosine monophosphate (AMP)-responsive element-binding protein H (CREBH) improves lipid metabolism and alleviates NAFLD lipotoxicity [15]. Furthermore, some studies have indicated that protein O-GlcNAcylation differentially influences hepatic metabolism and fibrosis [16,17]. Polyphenolic compounds, such as silibinin and curcumin, have reduced NAFLD/NASH by inhibiting O-GlcNAcylation in mouse models [18,19]. Therefore, it can be inferred that O-GlcNAcylation plays a critical role in the pathogenesis of NAFLD. 

The modification is also associated with various disorders related to abnormal glucose metabolism, including diabetic cardiomyopathy (DCM). Previous studies focused on the pathogenic mechanism of O-GlcNAcylation in DCM. Protein O-GlcNAcylation is significantly modified in the myocardium in diabetics and is a key regulator of the diabetic cardiac phenotype [20]. Mitigating this posttranslational protein modification improves DCM [21]. Interestingly, aberrant O-GlcNAcylation was detected in obesity, diabetes, cancer, and neurodegenerative diseases [22,23,24]. Also, the level of O-GlcNAcylation was upregulated in NASH mice [19]. In this review, O-GlcNAcylation in the pathogenesis of NAFLD is discussed and analyzed. Moreover, the application prospect of the intervention of O-GlcNAcylation in the treatment of NAFLD is reviewed for the first time.

## 2. Role of O-GlcNAc in Normal Liver Tissue

O-GlcNAcylation is a posttranslational modification requiring the attachment of a single O-linked β-N-acetylglucosamine (O-GlcNAc) moiety to the proteins [25,26,27]. The hexosamine biosynthetic pathway (HBP) regulates the O-GlcNAcylation levels. UDP-GlcNAc, a substrate for the protein O-GlcNAcylation, is produced in this process [28]. The two main enzymes involved in the regulation of protein O-GlcNAcylation modification are as follows: The O-GlcNAc transferase (OGT) catalyzes the transfer of a single N-acetylglucosamine to the proteins from UDP-GlcNAc, leading to their modification with the O-GlcNAc, and the single N-acetylglucosamine is hydrolyzed from the protein by O-GlcNAcase (OGA). O-GlcNAcylation has a reciprocal correlation with O-phosphorylation and modulates many biological processes in eukaryotes; thus, it is considered a critical regulatory modification [29]. 

**Figure 1:**
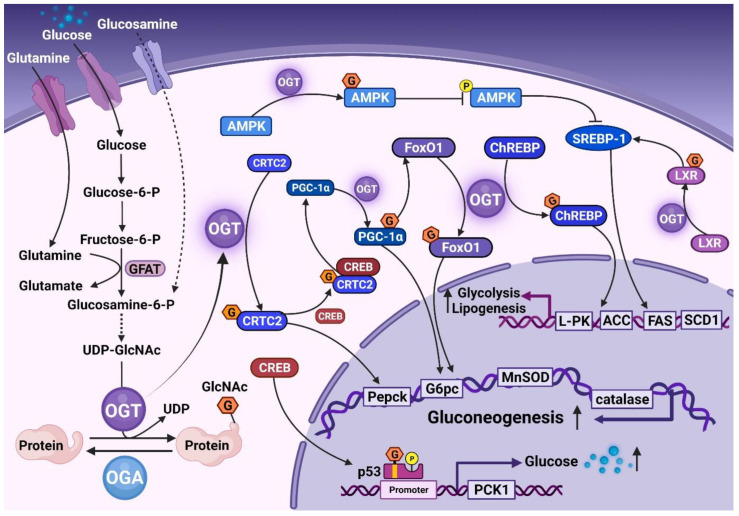
O-GlcNAcylation maintained normal physiological homeostasis in the liver. The HBP regulated the level of O-GlcNAcylation, and OGT catalyzed the transfer of single N-acetyl glucosamine from UDP-GlcNAC to the proteins; hydrolysis of a single N-acetylglucosamine from the proteins by OGA. O-GlcNAcylation of PGC-1α, FoxO1, and CRTC2 increases the expression of gluconeogenic genes and induces hepatic gluconeogenesis. O-GlcNAcylated p53 bound to the PCK1 promoter regulated the PCK1 levels and increased glucose synthesis. LXR, AMPK, ChREBP, and SREBP-1 were directly or indirectly regulated by O-GlcNAcylation, and subsequently, the transcriptional activity of target glycolysis and lipogenic genes was increased. HBP, hexosamine biosynthetic pathway; GFAT, glutamine fructose-6-phosphate amidotransferase; OGT, O-GlcNAc transferase; OGA, O-GlcNAcase; CRTC2, cAMP-regulated transcriptional co-activator 2; CREB, cyclic AMP-responsive element-binding protein; PGC1α, peroxisome proliferator-activated receptor-γ co-activator1α; ChREBP, carbohydrate-responsive element-binding protein; AMPK, AMP-activated protein kinase; SREBP-1, sterol regulatory element-binding protein 1; LXR, liver X receptors; ACC, acetyl-CoA carboxylase; FAS, fatty acid synthase; SCD1, stearoyl-CoA desaturase1; MnSOD, manganese superoxide dismutase.

O-GlcNAcylation is essential for maintaining the normal physiological homeostasis of the liver; studies have shown that modification acts as a metabolic sensor for liver clock regulation to maintain the circadian control of glucose [30,31]. Some studies have shown that O-GlcNAcylation plays a critical role in gluconeogenesis (Figure 1). The activity of peroxisome proliferator-activated receptor-γ co-activator1α (PGC1α) and FoxO1, key gluconeogenic regulators, is regulated by O-GlcNAcylation [32,33,34]. PGC1α, an essential coactivator of the transcriptional stimulation of gluconeogenic genes [35,36,37], further stimulates the expression of gluconeogenic genes. OGT affects PGC1α-mediated gluconeogenesis gene expression by targeting PGC1α via the host cell factor C1 (HCF-1) [34,35]. O-GlcNAcylation also stabilizes PGC1α by recruiting BAP1 for deubiquitination to promote gluconeogenesis [34]. PGC1α promotes OGT to effectuate O-GlcNAcylation and activate FoxO1 and increases the expression of Pepck and G6pc and the transcription of ROS detoxification enzymes, manganese superoxide dismutase (MnSOD) and catalase (CAT), further promoting hepatic glucose production [32]. OGT also increases the expression of Pepck and G6pc, which induces hepatic gluconeogenesis by the O-GlcNAcylation of the cAMP-regulated transcriptional co-activator 2 (CRTC2), a co-activator of the cyclic AMP-responsive element-binding protein (CREB) [38]. It has also been suggested that OGT is involved in glucocorticoid-induced gluconeogenesis [39]. p53 is usually recognized as a tumor suppressor [40]. A recent study reported that insulin sensitivity and liver glucose homeostasis are regulated by integrating the p53 signaling pathways, which depend on p53 O-GlcNAcylation. Subsequently, O-GlcNAcylated p53 binds to the PCK1 promoter to activate the gluconeogenic effect [41].

Furthermore, whether glucose flux promotes fat production through O-GlcNAcylation needed to be clarified. liver X receptors (LXRs) are lipid metabolism, glucose stability, and inflammation sensors. O-GlcNAcylation of the hepatic LXR was observed in refed mice and streptozotocin-induced diabetic mice [42]. High glucose increases the O-GlcNAcylation of the LXR and the transcriptional activity of the sterol regulatory element-binding protein 1 (SREBP-1) promoter. SREBP-1 is a master transcriptional regulator of hepatic lipogenesis [42], and the O-GlcNAcylation of the LXR upregulates the expression of SREBP-1 in the liver [42]. OGT regulates the phosphorylation and stability of SREBP-1 by increasing AMP-activated protein kinase (AMPK) O-GlcNAcylation in breast cancer [43], followed by the transcriptional activity of acetyl-CoA carboxylase (ACC) and fatty acid synthase (FAS). The carbohydrate-responsive element-binding protein (ChREBP) plays a vital role in glycolysis and lipogenesis. In hepatocytes, the O-GlcNAcylation of the ChREBP stabilizes the protein and increases its transcriptional activity on the target glycolysis [*liver pyruvate kinase* (*L-PK*)] and lipidogenic genes [*ACC*, *FAS*, and *stearoyl-CoA desaturase1* (*SCD1*)] [44]. Therefore, exploring the mechanistic and kinetic characterization of O-GlcNAcylation on key signaling proteins is promising for an in-depth understanding of normal hepatic metabolism. Finally, some studies have shown that multiple nodes of the insulin signaling pathway were altered by OGT. Under normal physiological conditions, O-GlcNAcylation is responsible for insulin signaling transduction. However, it would be abnormally elevated and induce IR in the state of overnutrition.

Elevated O-GlcNAcylation is not entirely detrimental to the liver. The termination of defective liver regeneration leads to reduced hepatocyte redifferentiation, severe necroinflammation, early fibrotic changes, and the formation of dysplastic nodules leading to the development of hepatocellular carcinoma (HCC) [45]. HNF4a O-GlcNAcylation in hepatocytes plays a key role in the termination of liver regeneration and prevention of hepatic dysplasia [45]. Studies have confirmed that calcium-dependent O-GlcNAc signaling is also critical in driving hepatic autophagy to maintain a nutrient and energy balance in response to starvation [46]. In addition, O-GlcNAc maintains a normal mitochondrial function, and the long-term elevation of o-GlcNAacylation coupled with an increased OGA expression modulates the mitochondrial function and reduces antioxidant responses [47]. For other liver diseases, such as hepatitis B, O-GlcNAcylation promotes the autophagic degradation of hepatitis B virus (HBV) replication virions and proteins through the mTORC1 signaling pathway and autophagosome-lysosome fusion, resulting in reduced HBV replication [48].

## 3. O-GlcNAcylation Contributes to IR

**Figure 2:**
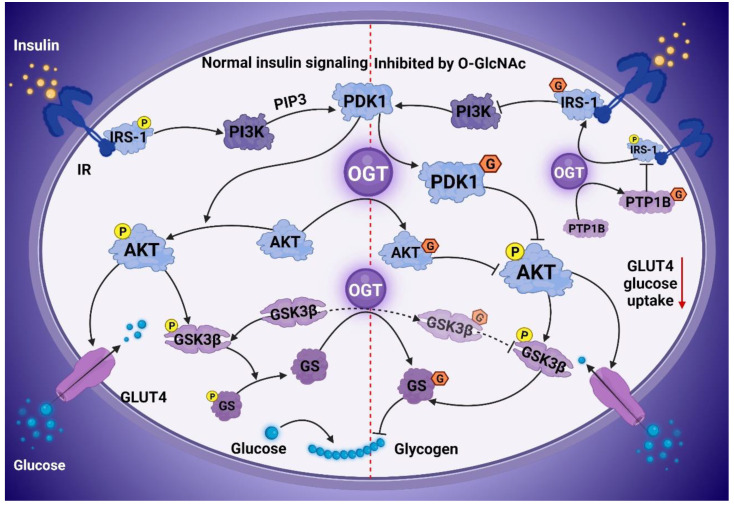
O-GlcNAcylation attenuated insulin signaling. Normal insulin signaling (Left). Insulin binding to the insulin receptor (IR) leads to the recruitment of IRS-1 and activates PI3K, producing PIP3 and activating PDK1 and AKT. The PI3K/AKT pathway induces the expression of GLUT4 and its transport from intracellular vesicles to cell membranes to promote glucose uptake. In addition, the PI3K/AKT pathway activates GSK3β and GS to promote glycogen synthesis. Insulin signaling was inhibited by O-GlcNAcylation (Right). OGT inactivated key insulin signaling proteins, including IRS-1, PI3K, PDK1, AKT, and PTP1B, and attenuated insulin signaling and insulin resistance. PI3K, phosphatidylinositol-3-kinase; PDK1, phosphoinositide-dependent protein kinase 1; AKT, serine/threonine-protein kinase B; GSK3β, glycogen synthase kinase 3 beta; GS, glycogen synthase; PTP1B, protein tyrosine phosphatase 1B; OGT, O-GlcNAc transferase.

The liver is an insulin-sensitive organ and essential for maintaining blood glucose. IR also plays a vital role in the occurrence and development of type 2 diabetes mellitus (T2DM) and NAFLD. Strikingly, NAFLD occurs in 70–80% of T2DM and obesity patients, and most NAFLD patients, develop hepatic IR [49]. The pathogenesis of NAFLD is closely related to IR as it is one of the components of the pathogenesis of NAFLD [50]. Additionally, IR is characterized by decreased glucose uptake and utilization in tissues, including liver tissue, adipose tissue, and muscle tissue [51]. IR increases the circulating free fatty acids through dysregulated lipolysis, resulting in an impaired insulin signal, a reduced clearance rate of glucose metabolism, and the dysregulation of lipid aggregation and decomposition [52]. In addition, the body increases lipid synthesis for energy by breaking down fat. Insulin increases lipase activity, thereby elevating the uptake of triglycerides by the adipose tissue and fat storage in the liver [53]. Lipid deposition in the liver further exacerbates IR. Preview studies demonstrated a critical role of O-GlcNAcylation in attenuating insulin signaling [49,50] (Figure 2). 

A major mechanism for terminating insulin signaling is the inactivation of insulin receptor substrates. OGT inactivates the insulin signaling proteins, including insulin receptor substrate 1 (IRS-1), phosphatidylinositol-3-kinase (PI3K), phosphoinositide-dependent protein kinase 1 (PDK1), serine/threonine-protein kinase B (AKT), and protein tyrosine phosphatase 1B (PTP1B), promoting the attenuation of insulin signaling [50,51]. Interestingly, OGT uses IRS-1 as its direct substrate [54]. In 3T3-L1 adipocytes, the Tyr608 phosphorylation of IRS-1 is inhibited by elevated O-GlcNAcylation, thereby reducing AKT activity [54]. In addition, OGT also uses PDK1 and PI3K as direct substrates in insulin signal attenuation [54,55]. Decreased AKT activity is vital for terminating insulin signaling, and O-GlcNAcylation plays a key role in the regulation of AKT activity. Normal O-GlcNAcylation is valuable for AKT signal transduction, while the O-GlcNAcylation of Thr305/Thr312 disrupts the interaction between AKT and PDK1, resulting in the downregulation of AKT activity [56]. Then, the decreased AKT activity reduces glycogen synthesis via glycogen synthase kinase 3 beta (GSK3β) phosphorylation. GSKβ is modified by O-GlcNAcylation, after the inhibition of GSK3β by lithium, and the overall O-GlcNAcylation level is significantly increased [57]. However, the function of GSK3β O-GlcNAcylation needs further exploration. In addition, PTP1B controls hepatic insulin signaling by inhibiting PTP1B O-GlcNAcylation, improving insulin sensitivity, and reducing liver lipid deposition [58]. Another study found that the O-GlcNAcylation level of glycogen synthase (GS) is increased, and the activity of GS is decreased after high glucose or glucosamine treatment, thereby leading to IR [59]. Therefore, abnormal glycogenogenesis and gluconeogenesis are closely related to O-GlcNAcylation during the development of hepatic IR.

## 4. Association of O-GlcNAc with NAFLD Process

**Figure 3:**
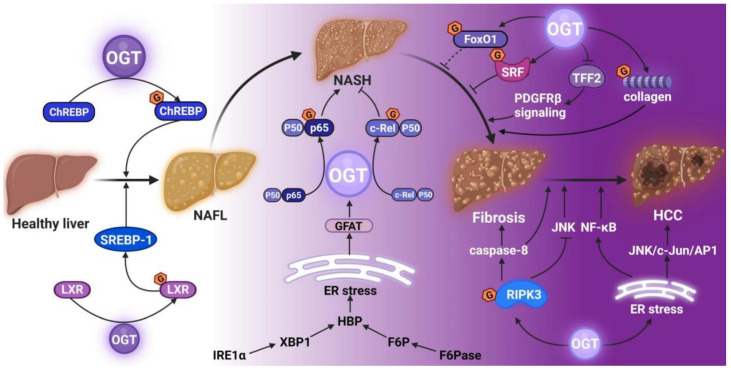
O-GlcNAcylation and NAFL-NASH-liver fibrosis-hepatoma tetralogy. The O-GlcNAcylation of the LXR, the ChREBP, and SREBP-1 promoted NAFL formation. O-GlcNAcylated NF-κB subunit p65 played a role in the progression of NASH by facilitating inflammatory damage, and the O-GlcNAcylated NF-κB subunit c-Rel exerted an anti-inflammatory effect. During liver fibrosis, O-GlcNAcylation of collagens accelerated fibrosis, while O-GlcNAcylation of the SRF represented anti-fibrotic effects. Finally, O-GlcNAcylated RIPK3 contributed to HCC. NAFL, non-alcoholic fatty liver; NASH, non-alcoholic steatohepatitis; LXR, Liver X receptor; carbohydrate-responsive element-binding protein; ChREBP, carbohydrate-responsive element-binding protein; SREBP-1, sterol regulatory element-binding protein 1; NF-κB, Nuclear factor-κB; SRF, serum response factor; HCC, hepatocellular carcinoma; OGT, O-GlcNAc transferase; GFAT, glutamine fructose-6-phosphate amidotransferase; ER stress, endoplasmic reticulum stress; HBP, hexosamine biosynthetic pathway; IRE1α, inositol requiring enzyme 1α; XBP1, X-box-binding protein 1; PDGFRβ, platelet-derived growth factor receptorβ; TFF2, trefoil factor 2; JNK, Jun N-terminal kinases.

O-GlcNAcylation acts as a promoting factor throughout NAFL-NASH-liver fibrosis-HCC. The LXR and the ChREBP are directly modified by O-GlcNAcylation, and SREBP-1 is indirectly regulated by O-GlcNAcylation, resulting in liver fat deposition and NAFL formation [42,43,44] (Figure 3). During the progression of NAFL to NASH, O-GlcNAcylation modifies I6PK1 and the Nuclear factor-κB (NF-κB) subunit p65 to increase the inflammatory injury, while the NF-κB subunit c-Rel undergoes O-GlcNAcylation to exert an anti-inflammatory effect under hyperglycemic conditions [17,60,61]. Moreover, the O-GlcNAcylation of collagens accelerates fibrosis, while that of the serum response factor (SRF) has an antifibrotic effect [62,63]. It modifies the receptor-interacting protein kinase 3 (RIPK3) to induce NAFLD-HCC [64,65].

### 4.1. O-GlcNAc and NAFL

NAFLD is a generalized term encompassing a range of liver conditions of varying severities resulting in liver fibrosis [52]; a simple steatosis named NAFL resulted from triglyceride accumulation in the cytoplasm of hepatocytes. On the other hand, the ChREBP is a pivotal transcription factor mediating the effects of glucose on glycolysis and lipogenesis genes. A previous study showed that the ChREBP is a regulatory center of adipogenesis in vivo and plays a decisive role in developing hepatic steatosis and IR; the specific inhibition of the ChREBP significantly improves hepatic steatosis in ob/ob mice [66]. A further study demonstrated that O-GlcNAcylation stabilizes the ChREBP and increases the activity on glycolytic lipogenic genes (*L-PK*, *ACC*, *FAS*, and *SCD1)* [44] (Table 1). Importantly, the overexpression of OGT significantly increases the ChREBP in C57BL/6J mice liver, resulting in enhanced lipogenic gene expression and excess hepatic triglyceride deposition [44]. Furthermore, HCF-1 O-GlcNAcylation, in response to glucose or a high-carbohydrate diet (HCD), first recruited OGT to the ChREBP, which led to ChREBP O-GlcNAcylation and activation [67]. Whether the mechanism of O-GlcNAcylation regulates the ChREBP in HCD-induced NAFLD mice needs to be investigated further.

The level of SREBP-1, a transcription factor that activates FAS and ACC1, is elevated [68], accompanied by hepatic steatosis [69]. Mice with the liver-specific overexpression of mature human SREBP-1 develop hepatic lipid accumulation and feature a fatty liver by the age of 6 months [70]. A previous study demonstrated that excessive glucose promotes lipid accumulation by upregulating lipid genes, such as *SREBP-1*, *FAS*, and *ACC1*, in cultured hepatocytes and animal model liver tissues [71]. Previous studies have shown that SREBP-1 protein expression is regulated by O-GlcNAcylation [43]. Also, the overexpression of glutamine fructose-6-phosphate amidotransferase (GFAT) promotes lipid accumulation in hepatic cells as well as inflammatory pathway activation by increasing the ER stress by the HBP [72], which indicates a critical role of the HBP in thyroglobulin (TG) accumulation. However, an updated study did not observe the response of SREBP-1 O-GlcNAcylation to GFAT inhibitors [73]. The correlation between SREBP-1 and the HBP and whether SREBP-1 directly effectuates O-GlcNAcylation is yet to be elucidated.

### 4.2. O-GlcNAc and NASH

In the preliminary stage, most patients with NAFLD manifest as hepatic steatosis without any symptoms. As the disease progresses, a proportion of the patients show NASH with inflammatory manifestation, hepatocyte injury, and fibrosis [74]. Nevertheless, the molecular mechanisms underlying the development of NAFLD and NASH are poorly understood. Protein O-GlcNAcylation impedes insulin signaling and promotes adipogenesis [16]. A recent study showed that inositol hexakisphosphate kinases 1 (IP6K1) inhibitors improve metabolic disorders, NAFLD/NASH. and fibrosis by altering these pathways [17]. How IP6K1 stimulates the protein O-GlcNAcylation to improve NAFLD by knocking down OGT remains to be explored.

Previous studies have indicated that O-GlcNAcylation is upregulated in NASH mice; however, the causal correlation between the upregulation of O-GlcNAcylation and the pathology of NASH is unclear. NF-κB, a proinflammatory transcription, is related to many pathogenic liver diseases [75], and NF-κB activated by inositol requiring enzyme 1α (IRE1α) causes liver inflammation and promotes NASH [76,77]. In addition, the activity of NF-κB is regulated by O-GlcNAcylation [60], and the upregulated O-GlcNAcylation activates NF-κB and increases inflammatory damage [78].

ROS accumulation and related ER stresses are caused by fat toxicity [79,80]. The transcription of GTAT is upregulated under ER stress, increasing protein O-GlcNAcylation [81]. Another study showed that O-GlcNAcylation, OGT, and GFAT levels are increased in mice with a methionine-choline deficient (MCD) diet, and the upregulated OGT and GFAT originate from the upstream target IRE1α induced via ER stress [19]. Currently, transcription factor X-box-binding protein 1 (XBP1) is the only known transcription factor downstream of IRE1α [82], and a key transcription factor is involved in hepatic adipogenesis and inflammation through ER stress [83]. These studies suggested that the upstream activator of the HBP is regulated by the transcription of XBP1 and is a positive regulatory loop for the onset of NASH. In another study, the expression of fructose-1,6-bisphosphatase (FBPase) was upregulated in NASH mice, leading to elevated F6P levels, HBP flux, and upregulated O-GlcNAcylation [18]. The increased level of protein O-GlcNAcylation by elevating the HBP flux in the liver plays a critical role in establishing a correlation between the increase in liver FBPase and NASH [84].

### 4.3. O-GlcNAc and Hepatic Fibrosis

Hepatic fibrosis is the most critical predictor of mortality in NAFLD, and the risk of liver-associated mortality increases exponentially with the increase in the fibrosis stage [85]. NASH patients with liver fibrosis are prone to develop cirrhosis [86]. Currently, only a few studies are related to O-GlcNAcylation and liver fibrosis. Hepatic stellate cells (HSCs) are the major source of the extracellular matrix in the liver [87]. Activated HSCs contribute to fibrogenesis. Interestingly, O-GlcNAcylation is involved in activating HSCs and collagen expression [62]. HSC activation originates from FoxO1 inactivation, leading to NAFLD fibrosis [88]. Paradoxically, the expression and activity of FoxO1 are increased in NASH patients [89]. Since FoxO1 plays a critical role in fibrosis and could be O-GlcNAcylated, it is essential to elucidate the role of FoxO1 O-GlcNAacylation on liver fibrosis through gene knockdown.

It was found that OGT-deficient hepatocytes are prone to hepatocyte ballooning, inflammation, and liver fibrosis [65]. OGT, a negative regulator of HSC activation, exerts a protective effect against hepatic fibrosis by boosting SRF O-GlcNAcylation. Therefore, the OGT expression and O-GlcNAcylation were decreased in HSCs isolated from MCD-fed mice livers [63]. In contrast, a recent study reported that OGT-deficient necroptotic hepatocytes secrete trefoil factor 2 (TFF2), which induces HSC activation, proliferation, and migration via platelet-derived growth factor receptorβ (PDGFRβ) signaling [90]. Thus, it is essential to clarify whether O-GlcNAc could be used as a biomarker for liver disease.

### 4.4. O-GlcNAc and NAFLD-HCC

NAFLD is becoming the leading cause of HCCs. NAFLD/NASH-HCC incidence and mortality rates are rising worldwide [91]. Furthermore, a retrospective cohort study from 2002 to 2012 indicated that NASH-related HCC increased significantly, and the number of patients undergoing liver transplantation for HCCs secondary to NASH increased by nearly four-fold, while the number of patients with HCCs secondary to chronic hepatitis C virus (HCV) increases only by two-fold [92]. NAFLD-HCC patients exhibit upregulated levels of OGT, which plays an oncogenic role by activating the oncogenic c-jun N-terminal kinases (JNK)/c-Jun/AP-1 and nuclear factor-kappa B (NF-κB) cascades [93]. Another study demonstrated that OGT is a key inhibitor of hepatocyte necroptosis in alcoholic fatty liver disease, and the lack of O-GlcNAcylation induces necroptosis in hepatocytes [65]. However, the specific pathogenesis mechanisms of NAFLD-HCC have not yet been totally revealed.

The mutual inhibition of caspase 8 and RIPK3 is essential for the development of NASH and hepatocarcinogenesis [64,94], and RIPK3 prevents cell proliferation from limiting the development of HCCs by inhibiting caspase 8 cleavage and JNK activation [64]. A study discovered that O-GlcNAcylation inhibits RIPK3 protein expression and stability [65]. Further investigation would analyze the molecular mechanism underlying OGT-regulated-*RIPK3* gene transcription by O-GlcNAcylation. Nonetheless, only a few studies have elaborated on the role of OGA in the liver. Targeting O-GlcNAcylation is a potential therapy for NAFLD-HCC.

Furthermore, OGT overexpression in the liver increased intracellular palmitic acid levels and promoted HCC by activating ER stress-associated oncogenic signaling cascades, including the JNK/c-Jun/AP1 and NF-κB signaling pathways [93]. Typically, 2/3 of NAFLD-HCC tumors show OGT overexpression, while 1/3 of no change in OGT expression is seen, suggesting that OGT expression is associated with gene polymorphism related to the occurrence and progression of NAFLD and NASH, such as *PNOLA3* p.I148M, *TM6SF2* p.E167K, and *MBOAT7* rs641738 [95,96]. Further studies should investigate whether OGT has a prognostic value for NAFLD-HCC.

## 5. Drugs Ameliorates NAFLD through Inhibition of O-GlcNAcylation

Metformin (MET) inhibits the proliferation of cervical cancer cells by reducing the O-GlcNAcylation of AMPK and increasing the level of phospho-AMPK [97] (Table 2). Another study indicated that MET inhibits the O-GlcNAc modification of NF-κB p65 and the ChREBP in the diabetic retina [98]. In addition, MET has been shown to have a protective effect on NAFLD, but the specific mechanism is yet unclear [99]. Furthermore, O-GlcNAcylation is activated, and AMPK/ACC pathway phosphorylation is inhibited in high-fat diet (HFD)-fed mice [100]. It has also been suggested that MET reduces hepatic TG accumulation and improves obesity-related NAFLD by inhibiting hepatic apolipoprotein A5 (ApoA5) synthesis through the AMPK/LXRα signaling pathway [101]. Therefore, it was speculated that MET promotes AMPK phosphorylation in the NAFLD liver by regulating AMPK O-GlcNAcylation and inhibiting the O-GlcNAc modification of the ChREBP, further increasing fat mobilization and reducing fat deposition in the liver. Also, inflammatory damage is alleviated by inhibiting the O-GlcNAc modification of NF-κB p65 in NAFLD patients.

The glucagon-like peptide-1 (GLP-1) receptor agonist, liraglutide, improves NASH by lowering liver enzyme levels and reducing liver fat [103]. Also, liraglutide and semaglutide improved NASH in clinical trials [114,115]. Yu et al. [116] proposed that GLP-1 inhibits the activation of the NLR family, pyrin domain-containing 3 (NLRP3) inflammasome, and reduced the production of ROS by enhancing mitophagy in hepatocytes, eventually improving NAFLD and delaying the progression of NASH. In addition, the activity of GLP-1 was enhanced by the inhibition of proteolysis due to O-GlcNAcylation [102]. However, the mechanisms underlying the elevated protein O-GlcNAcylation induced by GLP-1 that alleviated NAFLD/NASH are yet to be elaborated.

Goldberg et al., and Park et al. [117,118] speculated that increased O-GlcNAcylation enhances the pro-fibrotic signaling in mesangial cells exposed to high glucose. Sodium-glucose cotransporter 2 inhibitor (SGLT-2i) exerts antifibrotic effects in the diabetic kidney by reducing protein O-GlcNacylation [104]. In a clinical study, NAFLD patients treated with SGLT-2i experienced a remission of hepatic steatosis and improvement in liver fibrosis [105]. Some animal studies have also shown improvements in hepatic steatosis and steatohepatitis with various SGLT-2is, including remogliflozin, luseogliflozin, empagliflozin (EMPA), ipragliflozin, and NGI001 [119,120,121,122,123,124]. EMPA attenuated NAFLD in HFD-fed mice by activating autophagy and reducing ER stress and apoptosis [125]. Another study suggested that EMPA significantly improves NAFLD-related liver injury by enhancing the autophagy of hepatic macrophages through the AMPK/mammalian target of the rapamycin (mTOR) signaling pathway and further inhibiting the interleukin (IL)-17/IL-23 axis-mediated inflammatory response [126]. Presumably, SGLT-2i exerts an antifibrotic effect in NAFLD patients by reducing the protein O-GlcNacylation. It also ameliorates NAFLD/NASH by reducing ER stress and activates hepatocyte autophagy by inhibiting O-GlcNacylation.

The positive cardiovascular and metabolic effects of angiotensin (Ang)-converting enzyme inhibitors (ACEIs) are mainly dependent on the reduction of AngII formation and the increase in the negatively regulated Ang 1-7 axis of the renin-angiotensin system (RAS) [127,128]. Some studies have shown that ACE/AngII/AT1 contributes to the occurrence and progression of NAFLD [129]. The activation of the ACE2/Ang-(1-7)/Mas axis ameliorates hepatic IR through the Akt/PI3K/IRS-1/JNK insulin signaling pathway [130]. Moreover, Ang1-7 contributes to the correction of diabetic retinopathy by reducing the O-GlcNAcylation of the retinal protein in HFD-fed mice through the Mas/EPAC/Rap1/OGT signaling axis [106]. Also, ACEI therapy has been shown to reduce the incidence of liver cancer and cirrhosis in NAFLD patients [107].

Acetaminophen (APAP) overdose is a common cause of acute liver failure (ALF) in North American and European countries [131,132]. The increase in the hepatic O-GlcNacylated protein leads to the dysregulation of the hepatic glutathione (GSH) supplement response and increases the APAP-induced hepatic injury, while reduced O-GlcNacylation causes rapid GSH replenishment and the subsequent inhibition of APAP-induced liver injury [133]. Increased hepatic O-GlcNacylation as a response to excessive APAP increases and delays JNK activation, which is correlated to pronounced liver damage [133]. Moreover, Chen et al. [108] displayed a positive correlation between O-GlcNacylated c-Jun and GSH synthesis in clinical liver cancer samples. The overexpression of O-GlcNAcylated c-Jun inhibits ferroptosis by inducing GSH synthesis and blocking c-Jun O-GlcNacylation, which is beneficial for the treatment of iron apoptosis-related HCC [108]. Also, oral GSH exhibits a therapeutic effect on NAFLD patients; however, the mechanisms are remained unknown [109].

Alpha-lipoic acid (ALA) protects the kidney from oxidative damage in diabetic rats by reducing the O-GlcNAcylation of ERK and p38 [110]. In another study, ALA slowed the development of diabetic complications and ensured the function and health of red blood cells by reducing the O-GlcNAcylation modification levels of antioxidant enzymes: CuZn-superoxide dismutase (SOD), CAT, heat shock protein (HSP) 70, and HSP 90 [111]. Furthermore, it confirmed that the O-GlcNAcylation of the thioredoxin interacting protein (TXNIP) activates the NLRP3 inflammasome by interacting with the NLRP3 [134]. In a clinical trial, ALA was demonstrated to improve IL-6 and serum adiponectin levels in NAFLD patients [135]. Recently, two studies showed that ALA attenuates hepatic triglyceride accumulation and NAFLD by inhibiting the NLRP3 inflammasome [112,113]. Whether ALA plays a crucial role in NAFLD by changing the total level of O-GlcNAcylation or directly reducing the O-GlcNAcylation of NLRP3 and the role of O-GlcNAcylation in NAFLD, although drugs such as ALA, GSH, and ACEI exert a protective effect through anti-inflammatory and antioxidant effects, are yet to be clarified.

Hitherto, the pharmacological treatment of NAFLD by directly inhibiting O-GlcNAc has rarely been studied. Lee et al., showed that curcumin regulates the expression of SIRT1 and SOD1 through O-GlcNAcylation signaling [19]. It also reduces hepatitis by blocking the HBP flux signaling pathway; the anti-inflammatory effect of curcumin was achieved by inhibiting O-GlcNAcylation and blocking the NF-κB signaling pathway [19]. Silibinin blocks the NF-κB signaling pathway by inhibiting O-GlcNAcylation and alleviates inflammation in NASH mice [18]. Therefore, additional drug studies are required to further explore the treatment of NAFLD/NASH by targeting O-GlcNAcylation.

## 6. Conclusions

In this study, elevated O-GlcNAcylation promoted the development and exacerbation of IR and was eventually involved in the progression of NAFL-NASH-cirrhosis-hepatoma tetralogy. In addition, the potential drugs targeted at O-GlcNAcylation in the NAFLD intervention were reviewed. Thus, elucidating the molecular mechanisms of O-GlcNAcylation provided additional strategies and ideas for preventing and treating NAFLD.

## Figures and Tables

**Table 1:** Role of O-GlcNAc on the process of NAFLD.

Experiment Type	Key Factor	Directly Modified or Not	Level of O-GlcNAc	Specific Mechanism	Final Conclusion	Ref.
Animal and Cell	ChREBP	Yes	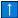	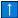 Transcriptional activity of L-PK, ACC, FAS, and SCD1	Hepatic TG deposition	[44]
Cell and Animal	SREBP-1	No	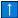	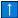 SREBP-1 phosphorylation and stability via AMPK signaling	TG deposition	[43]
Cell and Animal	IP6K1	No	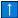	Unclarified	Promote NASH and fibrosis	[17]
Cell and Animal	NF-κB	Yes	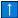	p65 is modified to induce activation of NFκB	Inflammatory damage	[60]
				c-Rel is modified and activated	Anti-inflammatory effect	[61]
Cell	Collagen	Yes	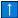	Activate HSCs	Liver fibrosis	[62]
Animal & Cell	SRF	Yes	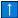	Inhibited SRF activity to induce α-SMA transcription	Prevent liver fibrosis	[63]
Animal & Cell	RIPK3	Yes	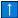	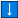 RIPK3 stability, caspase 8 cleavage, and JNK activation	Promote NAFLD-HCC	[64,65]

ChREBP, carbohydrate-responsive element-binding protein; L-PK, liver pyruvate kinase; ACC, acetyl-CoA carboxylase; FAS, fatty acid synthase; SCD1, stearoyl-CoA desaturase1; TG, thyroglobulin; SREBP-1, sterol regulatory element-binding protein 1; AMPK, AMP-activated protein kinase; IP6K1, inositol hexakisphosphate kinases 1; NASH, non-alcoholic steatohepatitis; NF-κB, Nuclear factor-κB; HSCs, hepatic stellate cells; SRF, serum response factor; α-SMA, α-smooth muscle actin; RIPK3, receptor-interacting protein kinase 3; JNK, c-Jun N-terminal kinases; NAFLD-HCC, NAFLD-associated hepatocellular carcinoma. Up arrow represents up-regulation, Down arrow represents down-regulation.

**Table 2:** Drug interactions with O-GlcNAcylation and NAFLD.

Drug.	Correlation with O-GlcNAcylation	Effects on NAFLD	Ref.
MET	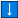 O-GlcNAcylation of AMP, NF-κB, and ChREBP	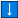 Liver TG accumulation and improved NAFLD	[97,98,99,101]
GLP-1	O-GlcNAcylation enhance GLP-1 activity	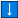 Liver enzyme levels and liver fat	[102,103]
SGLT-2I	Reduced O-GlcNAcylation exerts an anti-fibrotic effect	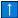 Liver steatosis and liver fibrosis	[104,105]
ACEI	Enhancement of Ang1-7 axis to reduce O-GlcNAcylation	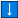 Incidence of liver cancer and cirrhosis	[106,107]
GSH	Positive correlation between c-Jun O-GlcNAcylation and GSH synthesis	supported liver metabolism and improved NAFLD	[108,109]
ALA	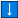 O-GlcNAcylation of ERK, p38, CuZnSOD, CAT, HSP70, and HSP90	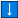 Liver TG accumulation and improved NAFLD	[110,111,112,113]
Curcumin	Inhibition O-GlcNAcylation and blocked NF-κB signaling pathway	Exert anti-inflammatory effect, alleviated NAFLD/NASH	[19]
Silibinin	Inhibition of O-GlcNAcylation and blocked NF-κB signaling pathway	Anti-inflammatory effect, alleviated NASH	[18]

NAFLD, non-alcoholic fatty liver disease; MET, metformin; AMP, cyclic adenosine monophosphate; NF-κB, Nuclear factor-κB; ChREBP, carbohydrate-responsive element-binding protein; GLP-1, glucagon-like peptide-1; SGLT-2I, sodium-glucose cotransporter 2 inhibitor; Ang, angiotensin; ACEI, Ang converting enzyme inhibitors; GSH, glutathione; ALA, alpha-lipoic acid; CuZnSOD, CuZn-superoxide dismutase; CAT, catalase; HSP, heat shock proteins. Up arrow represents up-regulation, Down arrow represents down-regulation.

## Abbreviations

Ang	angiotensin
APAP	acetaminophen
ALF	acute liver failure
ALA	alpha-lipoic acid
ApoA5	apolipoprotein A5
ACC	acetyl-CoA carboxylase
AMP	adenosine monophosphate
AMPK	AMP-activated protein kinase
AKT	serine/threonine-protein kinase B
ACEI	Angiotensin-converting enzyme inhibitors
CAT	catalase
CRTC2	cAMP-regulated transcriptional co-activator 2
CREB	cyclic AMP-responsive element-binding protein
CREBH	cyclic AMP-responsive element-binding protein H
ChREBP	carbohydrate-responsive element-binding protein
DCM	diabetic cardiomyopathy
EMPA	empagliflozin
ER	endoplasmic reticulum
FAS	fatty acid synthase
FBPase	fructose-1,6-bisphosphatase
GSH	glutathione
GS	glycogen synthase
GLP-1	glucagon-like peptide-1
GSK3β	glycogen synthase kinase 3 beta
GFAT	glutamine fructose-6-phosphate amidotransferase
HCV	hepatitis C virus
HSP	heat shock protein
HCF-1	host cell factor C1
HSCs	hepatic stellate cells
HCD	high-carbohydrate diet
HCC	hepatocellular carcinoma
HBP	hexosamine biosynthetic pathway
IL	interleukin
IR	insulin resistance
IRS-1	insulin receptor substrate 1
IRE1α	inositol requiring enzyme 1α
IP6K1	inositol hexakisphosphate kinases 1
JNK	Jun N-terminal kinases
LXRs	liver X receptors
L-PK	liver pyruvate kinase
MET	metformin
MCD	methionine-choline deficient
mTOR	mammalian target of rapamycin
MnSOD	manganese superoxide dismutase
MAFLD	metabolic dysfunction-associated fatty liver disease
NASH	non-alcoholic steatohepatitis
NAFLD	non-alcoholic fatty liver disease
NLRP3	NLR family, pyrin domain containing 3
OGA	GlcNAcase
OGT	O-GlcNAc transferase
O-GlcNAc	O-linked β-N-acetylglucosamine
PI3K	phosphatidylinositol-3-kinase
PTP1B	protein tyrosine phosphatase 1B
PDGFRβ	platelet-derived growth factor receptorβ
PDK1	phosphoinositide-dependent protein kinase 1
PGC1α	peroxisome proliferator-activated receptor-γ co-activator1α
ROS	reactive oxygen species
RAS	renin-angiotensin system
RIPK3	receptor-interacting protein kinase 3
SRF	serum response factor
SOD	superoxide dismutase
SCD1	stearoyl-CoA desaturase1
SGLT-2i	sodium-glucose cotransporter 2 inhibitor
SREBP-1	sterol regulatory element-binding protein 1
TG	thyroglobulin
TFF2	trefoil factor 2
T2DM	type 2 diabetes mellitus
TXNIP	thioredoxin interacting protein
XBP1	X-box-binding protein 1
